# Melanin Concentration Gradients in Modern and Fossil Feathers

**DOI:** 10.1371/journal.pone.0059451

**Published:** 2013-03-26

**Authors:** Daniel J. Field, Liliana D’Alba, Jakob Vinther, Samuel M. Webb, William Gearty, Matthew D. Shawkey

**Affiliations:** 1 Department of Geology and Geophysics, Yale University, New Haven, Connecticut, United States of America; 2 Department of Biology and Integrated Bioscience Program, University of Akron, Akron, Ohio, United States of America; 3 Departments of Earth and Biological Sciences, Bristol University, Bristol, United Kingdom; 4 Stanford Linear Accelerator Center, Menlo Park, California, United States of America; University of Lausanne, Switzerland

## Abstract

In birds and feathered non-avian dinosaurs, within-feather pigmentation patterns range from discrete spots and stripes to more subtle patterns, but the latter remain largely unstudied. A ∼55 million year old fossil contour feather with a dark distal tip grading into a lighter base was recovered from the Fur Formation in Denmark. SEM and synchrotron-based trace metal mapping confirmed that this gradient was caused by differential concentration of melanin. To assess the potential ecological and phylogenetic prevalence of this pattern, we evaluated 321 modern samples from 18 orders within Aves. We observed that the pattern was found most frequently in distantly related groups that share aquatic ecologies (e.g. waterfowl Anseriformes, penguins Sphenisciformes), suggesting a potential adaptive function with ancient origins.

## Introduction

Complex pigmentation patterns like spots and stripes are common features of avian plumage [1], [2], and their evolution, development, heritability, and functionality have received considerable attention [3], [4], [5], [6]. Recently, the discovery of fossilized melanosomes (melanin-containing organelles) has allowed identification of highly contrasting and conspicuous patterning between and within fossil feathers [1]. Analogous patterns in modern birds have led to the suggestion that sexual display played a critical role in the early evolution of feathers [1], [7]. Less striking patterns that may be important for other reasons (e.g. mottling for crypsis) have not yet been identified in fossils.

A fossil feather with a distinct gradient of coloration along the proximo-distal axis was recovered from the Fur formation in Denmark [8]. The same gradient is observed on both the part and counterpart of the fossil, demonstrating that its presence is not an artifact of uneven fossil splitting (Figure S1). To our knowledge, the proximate basis of this pattern, as well as its distribution within birds, was unknown. Thus, to gain some understanding of this pattern, and to assess if melanosome concentration can be used to infer relative color intensity of fossil feathers, we tested if the gradient was caused by differential distribution of melanosomes using (1) keratin removal and light microscopy on extant feathers, and (2) scanning electron microscopy (SEM). We also subjected the fossil to a recently developed trace metal mapping technique [9]. Finally, we examined the distribution of this gradient in modern birds as a first step towards examining its potential function.

## Materials and Methods

### Assessing Melanosome Concentration in Modern Feathers

Based on relationships between melanin concentration and darkness in human hair [10], we hypothesized that a decreasing density of melanosomes caused the observed gradient from the distal to the proximal end of the feather. To test this hypothesis, dark feathers from study skins of five extant taxa (Phalacrocorax auritus, Branta canadensis, Fratercula arctica, Himantopus mexicanus and Larus atricilla), were placed between two glass microscope slides. A 10% solution of Na_2_S (Acros, Morris Plains, NJ), which dissolves feather keratin by breaking disulfide bridges of cystine [11], was injected between the slides to remove the keratin matrix surrounding the melanosomes. Melanin does not contain disulfide bridges, and is a tough and insoluble polymer [12]; thus, it should be unaffected by the treatment, despite the complete degradation of keratin. Enough solution was used to wet the entire feather, and samples were incubated at 40°C for 4 hours. For large feathers a second coating with Na_2_S was performed to ensure keratin degradation to the point where melanosomes, but not feather keratin, were observed. After a maximum of 6 hours of incubation, the Na_2_S solution was carefully rinsed away by slowly injecting distilled water through the slides as before. After drying, the two slides were separated, leaving prints of melanosomes on both slides (Figure S2), which were examined with light microscopy. Grey values of the original feathers were determined in ImageJ (available at http://rsb.info.nih.gov/ij; developed by Wayne Rasband, National Institutes of Health, Bethesda, MD), and melanosome concentration was quantified on both slides using ImageJ’s ‘count particles’ function from light micrographs of the dissolved feathers.

### Fossil SEM

**Figure 1:**
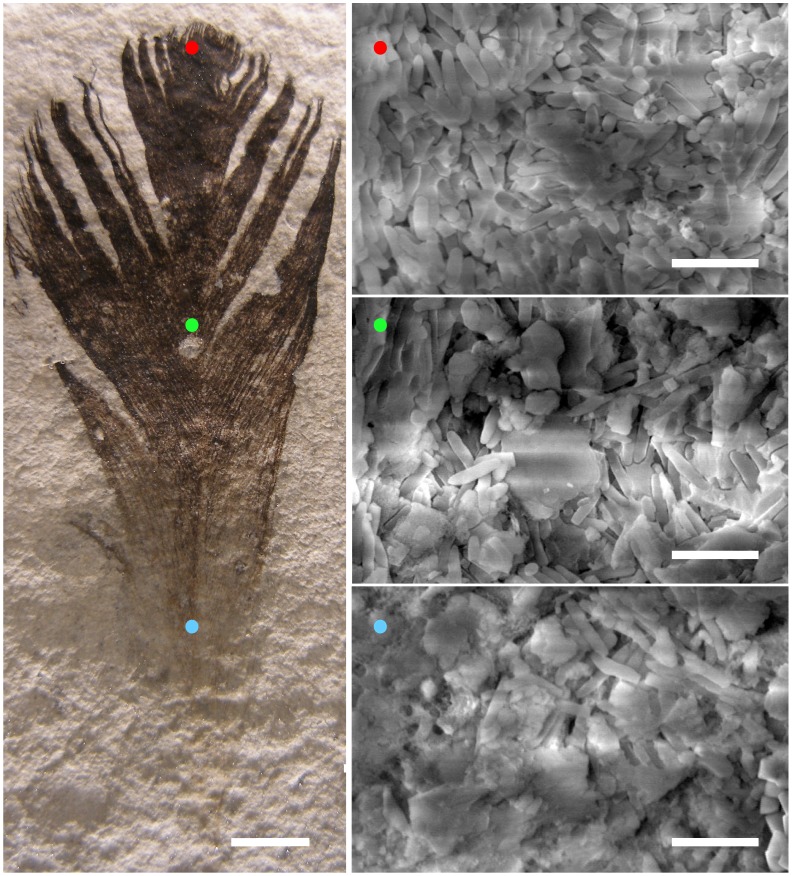
Representative SEM micrographs from the distal (red), middle (green), and proximal (blue) portions of a ∼55 myr fossil bird feather from the Danish Fur Formation. In the micrographs, rod-shaped structures are melanosomes; the concentration of these melanin-containing organelles decreases from the distal to the proximal end of the feather (see Fig. 2). For the fossil, scale bar represents 5 mm. In the SEM micrographs, scale bars represent 2 µm.

We then tested if the relationship between melanosome density and color held true in the fossil feather (Moler Museet, Fur, Denmark, 5-1003) using two methods. First, we placed a copper wire mesh on top of the feather (the part), dividing it longitudinally into five equal-width 6.2 mm bins. Tin foil was wrapped around the sides and bottom of the fossil to reduce charging in the scanning electron microscope. Twenty images were taken within each bin at randomized locations along the proximal end of barb rami using a Philips XL 30 ESEM (Fig. 1). Melanosome concentration was determined by counting melanosomes in each scanning electron micrograph, and grey value was determined using ImageJ.

### Fossil Trace Metal Mapping

Second, we used the trace metal mapping techniques developed and described in [9]. Their data suggest that several trace metals, including copper and zinc, which are chelated by melanin [13], can be used as proxies for the presence and abundance of eumelanin. We note that other fossil organic materials are known to chelate copper and other metal ions, such as humic acids and geoporphyrins (e.g. [14]), so this method alone cannot confirm the presence or preservation of melanin. However, we used the following methods to produce trace metal maps of the feather (the part) to corroborate our SEM observations.

X-ray fluorescence trace metal mapping images were collected at the Stanford Synchrotron Radiation Lightsource (SSRL) using beam line 10–2. The incident x-ray energy was set to 11.0 keV using a Si (111) double crystal monochromator with the storage ring Stanford Positron Electron Accelerating Ring (SPEAR) containing 350 mA at 3.0 GeV. The fluorescence lines of the elements of interest, as well as the intensity of the total scattered X-rays, were monitored using a silicon drift Vortex detector (SII NanoTechnology USA Inc.). The microfocused beam of ∼50×50 µm was provided by a focusing x-ray polycapillary optic (XOS). The incident and transmitted x-ray intensities were measured with nitrogen-filled ion chambers. Samples were mounted at 45° to the incident x-ray beam and were spatially rastered in the microbeam while data were collected continuously during stage motion. Beam exposure was 30 ms per pixel. Metal concentrations (µg cm^−2^) were calibrated from the dead time corrected fluorescence counts in each channel by using thin film standards deposited on mylar from Micromatter (Vancouver, Canada), and collected under the same conditions of beam intensity and sample to detector distance. Data processing consisted of taking the median intensity over a 3×3 pixel area which improved signal to noise ratios over areas with lower count rates. We calculated mean metal concentrations in fifteen different regions of interest (ROIs) throughout the feather and in the surrounding matrix (see Figure S3 for sampling locations).

### Classification of Within-feather Pigmentation Gradients

To assess the prevalence of within-feather pigmentation gradients in modern birds, we examined 321 black contour and flight feathers from 60 different families (18 orders), from the United States National Museum collection. Two independent observers (authors MDS and LD) graded feathers on a scale of zero to three, with zero indicating absence of the gradient, 1 indicating presence of the gradient, 2 indicating a binary pigment deposition with a uniformly dark pennaceous feather and an unpigmented afterfeather (downy basal part of the feather vane), and 3 indicating an inverse gradient (pigmentation decreasing from proximal to distal end of feather). Examples of these gradient categories are depicted in Figure S4. Data are tabulated in Table 1.

**Table 1:** Clade-specific frequency of melanin concentration gradients in feathers.

Order	Percentage of feathers with color gradient 1	Number of feathers sampled
Sphenisciformes	100.0	10
Anseriformes	88.0	25
Gaviiformes	87.5	8
Charadriiformes	69.2	13
Podicipediformes	68.8	16
Falconiformes	66.7	6
Suliformes	57.1	21
Procellariiformes	50.0	10
“core-Gruiformes”	47.8	23
Ciconiiformes	35.7	14
Accipitriformes	29.6	27
Coraciiformes	25.0	16
Apodiformes	24.0	25
Columbiformes	19.0	21
Cuculiformes	11.1	18
Passeriformes	7.8	51
Tinamiformes	0.0	7
Piciformes	0.0	10

Names in bold denote clades of birds with largely aquatic ecologies.

## Results

**Figure 2:**
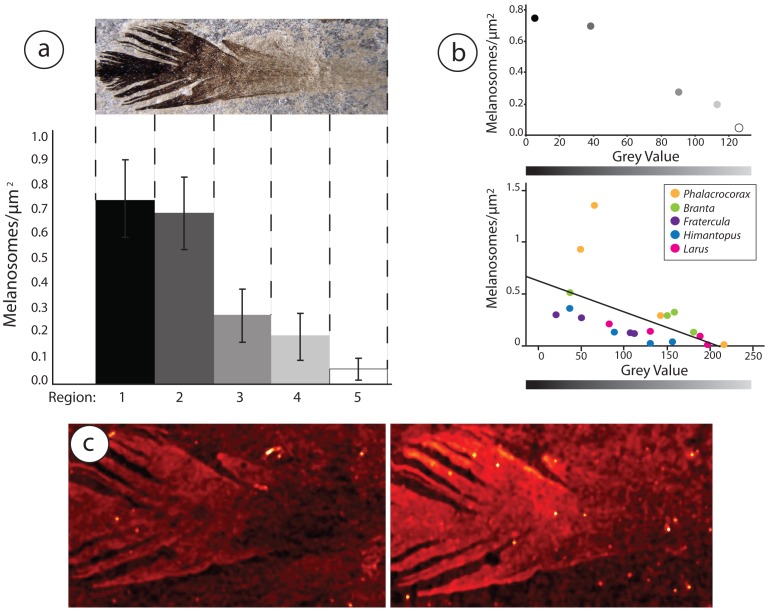
Correlations between melanosome gradients and feather darkness. A–B) Melanosome concentrations in darker and lighter sections of fossil (a, top of b) and extant (bottom of b) feathers. C) False color images of copper (left) and zinc (right) concentrations in the fossil feather.

Spearman’s rank analysis (Fig. 2B) showed a significant relationship between melanosome concentration and feather grey value in extant feathers (ρ = −0.069; p = 0.001). Similarly, melanosome concentration of the fossil decreased significantly from the distal tip of the feather to its proximal end (Fig. 2A); this was not due to taphonomic artifact, as the feather’s melanosomes lie on top of the matrix along its entire distoproximal length. No melanosomes were detected in the matrix surrounding the feather (Figure S3). Grey value decreases approximately 10-fold over this distance (Fig. 2B). Additionally, copper and zinc levels associated with melanin deposition also strongly correlate with darkness (Fig. 2C), further supporting our hypothesis that the gradient is attributable to differential melanization. Overall, we found reasonably good agreement between concentration patterns detected by SEM and trace metal mapping (Fig. 2). However, it is worth noting that the latter detected no melanin in the most basal portions of the feather despite its presence as indicated by melanosomes in SEM images (Fig. 1, Table S1). This discrepancy indicates that trace metal mapping lacks some sensitivity, and that its results should always be verified by additional methods.

Within-feather pigmentation gradients are observed in 38% of sampled extant feathers; the inverse gradient (dark proximal, white distal) is observed in 3%. The standard gradient was found in contour feathers with higher frequency than in flight feathers (x^2^ = 5.12, p = 0.02). A bimodal pigmentation pattern (black feather, white afterfeather) was found in an additional 15% of samples. No pattern was observed in the remaining 44% of samples. Interestingly, the occurrence of within-feather pigmentation gradients seems to be associated with ecology, as gradients are most commonly observed in waterbirds that are distantly related to one another (Table 1). For example, it is found in 100% of penguins sampled and in 88% of waterfowl, but only in 7.8% of passerines.

## Discussion

Discrete color patterns of feathers have been studied [2], [3], but more subtle patterns have not. Here we show that an unstudied pigmentation pattern is caused by a melanosome concentration gradient in both fossil and extant feathers, and is found largely in groups of extant birds sharing aquatic ecologies.

By quantitatively demonstrating a link between feather melanosome concentration and feather color, we help substantiate the common assumption that darker plumage can be caused by the deposition of larger amounts of melanin (e.g. [15], [16]). These data are needed to validate discussion of, for example, the potential physiological costs of producing darker plumage [12], and the inference of color gradients from darkness gradients in fossil feathers [17]. Our data show that melanosome density predicts brightness of some melanin-based colors, suggesting that it can be used to help determine if feathers were originally dark or pale. These data may help improve the resolution of fossil color reconstructions, enabling more precise functional inferences, and the quantification of intraspecific variation. Because sexual dimorphism [18], [19] and age [20], [21] may influence melanin-based colors of some species, this latter ability may potentially enable detection of sexual dichromatism and individual maturation in the fossil record. Finally, by providing a means to estimate relative pigmentation (quantifying melanosome concentration within feathers), we demonstrate a simple method for estimating the degree of feather melanization that could be useful in studies of, for example, plumage color signaling in both fossil and modern feathers. This method of assessing relative color intensity by means of quantifying melanosome concentration is attractive, as the mode and quality of fossil feather preservation varies greatly, and in some cases only melanosome impressions remain [1], [22], [7]. However, although they are likely closely linked, future work should use additional chemical methods (e.g. [23]) to verify how closely melanosome density is correlated with melanin concentration.

The question of why a feather is colored a certain way can be answered at both a proximate and ultimate level [24]. Thus, our data raise the question of why within-feather pigment concentration gradients exist. A first hypothesis is based on the idea that melanin deposition may be costly. Both eumelanin and phaeomelanin are produced from L-tyrosine via a complex biochemical mechanism, which may be associated with significant energetic costs [12]. Piault *et al*. report condition-dependent expression of melanin in the Eurasian Kestrel (*Falco tinnunculus*), corroborating the assumption that melanin expression may be costly [25]; however, Roulin *et al*. found no evidence for condition dependence of melanin-based ornamentation in an identical study of Barn Owls (*Tyto alba*) [26]. Since only the distal ends of feathers are usually exposed to the external environment (the proximal ends are obscured by surrounding feathers), darkly pigmented distal feather tips may also allow the feather patch to appear fully black, despite the fact that color saturation steadily declines towards a feather’s proximal end. Since feathers may be melanized for coloration, to provide enhanced resistance to mechanical abrasion, or to combat deterioration by feather-degrading bacteria [27], [28], this pattern allows the exposed portion of the feather to be melanized, while minimizing melanization in regions that will neither be seen, nor encounter abrasion from environmental or microbial sources [29]. Supporting this interpretation, distal barbules, which lie on top of proximal barbules and are therefore the more visible of the two [30], tend to be more darkly pigmented than proximal barbules [31]. However, the physiological cost of melanin production is debatable [12], and moreover, this hypothesis predicts that the vast majority of dark feathers, and not the 38% found here, should exhibit pigmentation gradients.

Alternatively, the high frequency of pigmentation gradients and their conservation in numerous distantly related waterbird clades suggest that these gradients may serve an adaptive ecological function. Such convergent evolution in animals with shared ecologies typically indicates adaptation and/or constraint [32]. The plumulaceous vane helps trap air between the plumage and skin, increasing both heat retention and buoyancy. Melanin is known to enhance feather stiffness [27], but this is an undesirable property in downy feathers whose barbs should lie loosely across one another to trap air [30]. Thus, decreasing the amount of melanin at a feather’s base may allow for a less stiff afterfeather, and hence potentially greater heat retention and buoyancy. The former may be particularly important in penguins, as most of the insulating properties of their contour feathers derive from their plumulaceous afterfeathers [33]. Other aquatic birds, which may or may not encounter such cold temperatures, will still depend on buoyancy regulation that may be enhanced by a loose afterfeather. This hypothesis should be tested through future thermal and buoyancy measurements.

These data suggest that within-feather pigmentation gradients are relatively common amongst birds, and are found most frequently in waterbirds. Additionally, we demonstrate that melanosome concentration reflects feather brightness, and thus may help improve the discriminant power of fossil feather color reconstructions. The gradients seen in dark feathers lacking other within-feather pigmentation patterns [2] may reflect selection acting to minimize production of metabolically costly melanin, or minimize proximal melanin deposition to reduce the stiffness of downy afterfeathers. The presence of this pattern in the Eocene feather here, as well as in *Archaeopteryx* [17], suggest that the importance of this pressure may be as ancient as the avialan clade itself.

## Supporting Information

Figure S1 **Part (left) and counterpart (right) of a fossil feather recovered from the Fur Formation of Denmark.** Identical darkness gradients can be observed in both, ruling out uneven splitting as the cause of the gradient observed in the part.
(TIFF)

Figure S2 **The process of Na_2_S feather degradation, and subsequent melanosome density analysis.** An intact feather is placed between two glass microscope slides, and wetted with Na_2_S. After an incubation period and rinsing, the slides are separated to reveal a melanin print on both slides. Light micrographs of the melanin print are then analyzed to quantify differential melanosome densities in various regions of the feather. A) Intact contour feather from *Himantopus mexicanus,* B) Same feather after treatment with Na_2_S, removing most of the keratin surrounding the melanin, C) and D) close ups of dark (C) and light (D) regions of the feather showing higher concentrations of melanosomes in the dark region. Scale bars = 10 µm
(PDF)

Figure S3 A) Locations of 15 regions of interest (ROIs), where mean metal concentrations were analyzed. B) Representative image of matrix surrounding the fossil feather, demonstrating that no melanosomes are present.
(PDF)

Figure S4 **Representative pictures of feathers exhibiting the four pigment concentration gradient categories.** (0) absence of gradient (*Ramphastos tucanus*), (1) presence of gradient (*Aechmophorus occidentalis),* (2) binary gradient (*Sarcoramphus papa*), (3) inverse gradient (*Fluvicola nengeta*).
(PDF)

Table S1 **Average trace metal concentration for the 15 regions of interest (ROIs) shown in Figure S2, covering various portions of the feather, and the surrounding matrix.**
(XLSX)
