# Supplementary figures and images for: Melanin Concentration Gradients in Modern and Fossil Feathers
Source: PLoS One. 2013 Mar 26;8(3):e59451. doi: 10.1371/journal.pone.0059451 (PMC3608712; doi:10.1371/journal.pone.0059451)

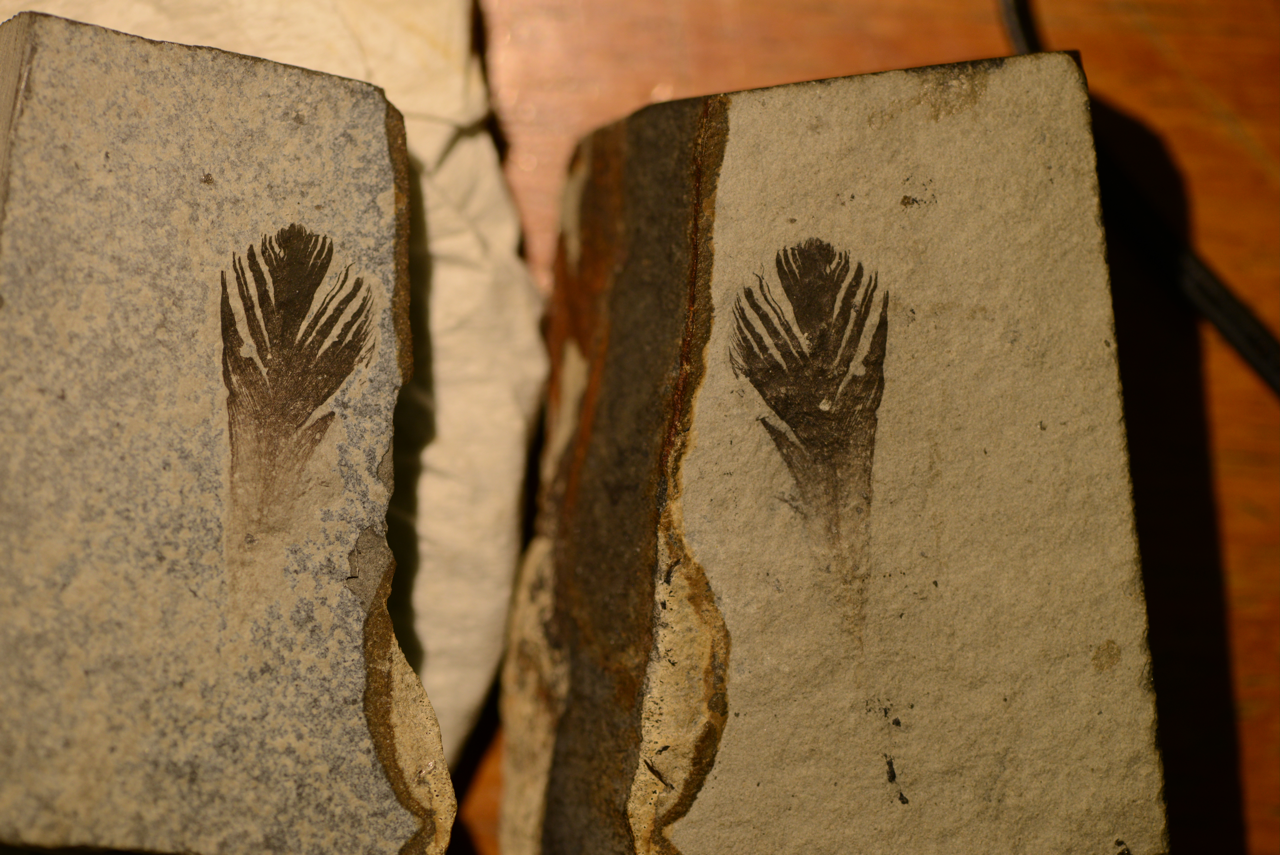

Supplement: Figure S1 — Part (left) and counterpart (right) of a fossil feather recovered from the Fur Formation of Denmark. Identical darkness gradients can be observed in both, ruling out uneven splitting as the cause of the gradient observed in the part. (TIFF) [file pone.0059451.s001.tiff]

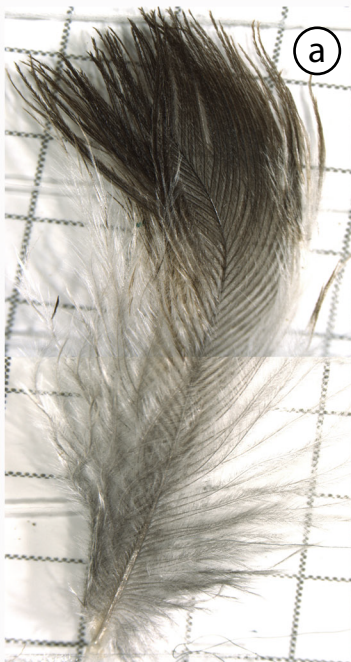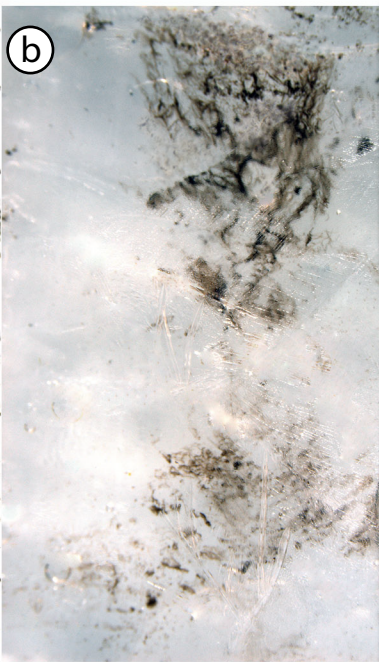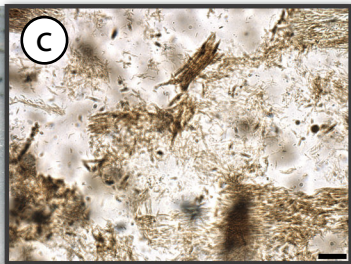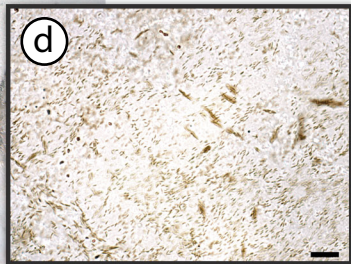

Supplement: Figure S2 — The process of Na2S feather degradation, and subsequent melanosome density analysis. An intact feather is placed between two glass microscope slides, and wetted with Na2S. After an incubation period and rinsing, the slides are separated to reveal a melanin print on both slides. Light micrographs of the melanin print are then analyzed to quantify differential melanosome densities in various regions of the feather. A) Intact contour feather from Himantopus mexicanus, B) Same feather after treatment with Na2S, removing most of the keratin surrounding the melanin, C) and D) close ups of dark (C) and light (D) regions of the feather showing higher concentrations of melanosomes in the dark region. Scale bars = 10 µm (PDF) [file pone.0059451.s002.pdf]

a

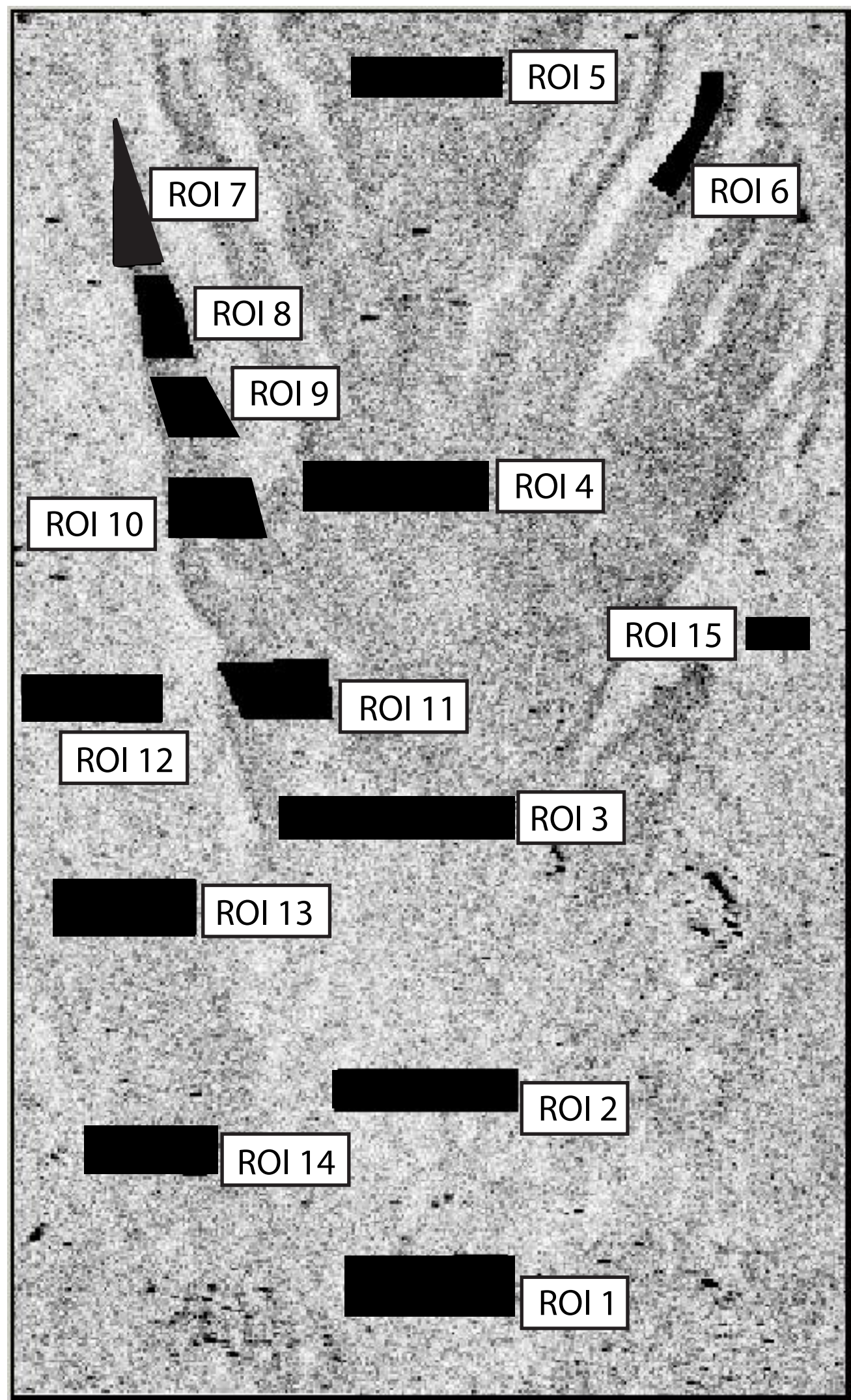

b

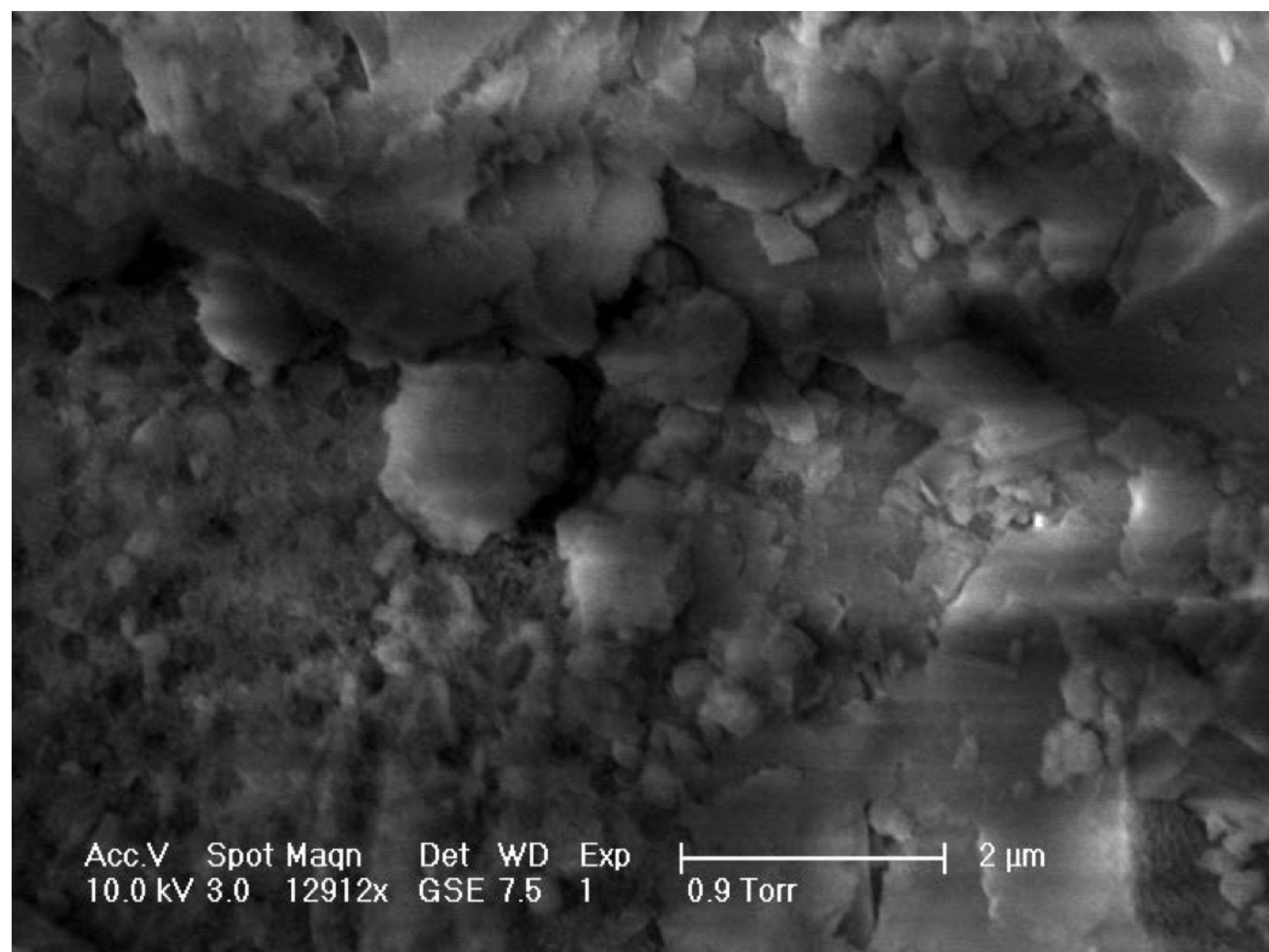

Supplement: Figure S3 — A) Locations of 15 regions of interest (ROIs), where mean metal concentrations were analyzed. B) Representative image of matrix surrounding the fossil feather, demonstrating that no melanosomes are present. (PDF) [file pone.0059451.s003.pdf]

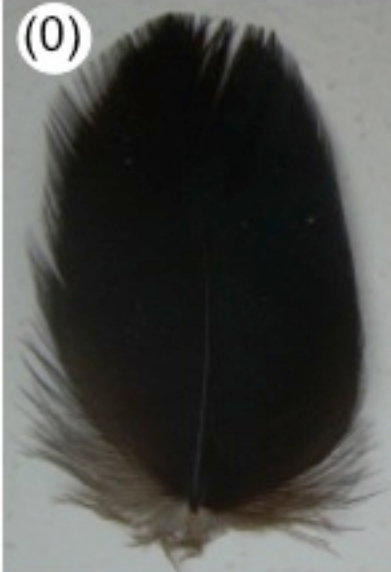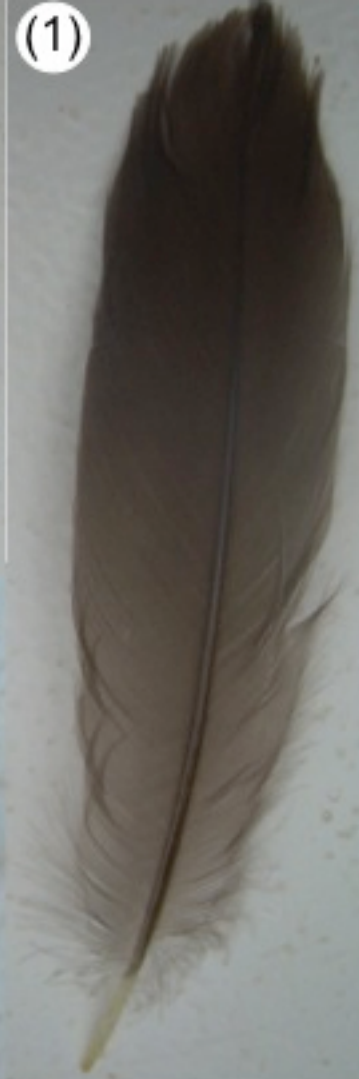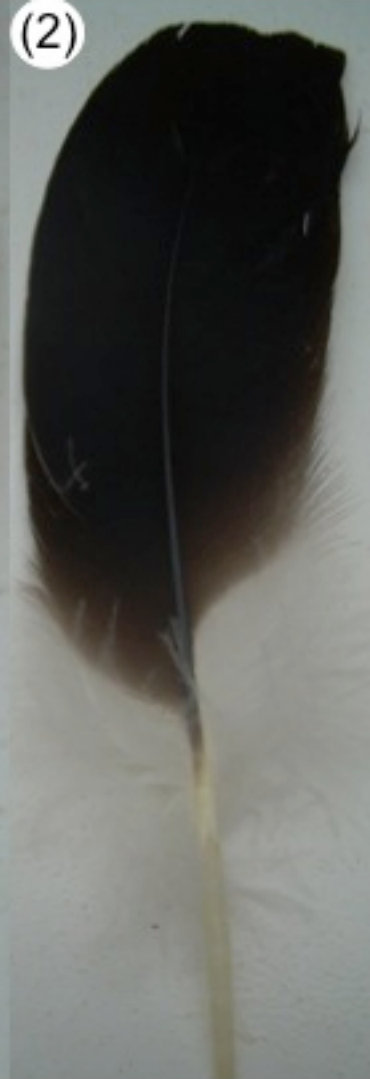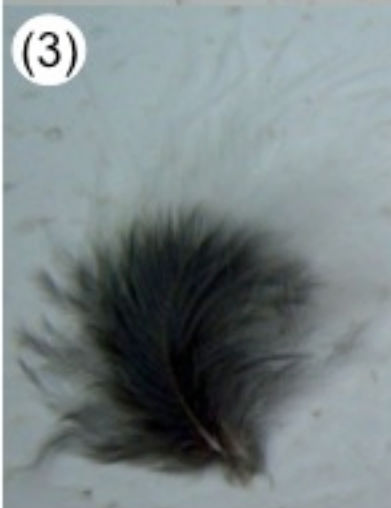

Supplement: Figure S4 — Representative pictures of feathers exhibiting the four pigment concentration gradient categories. (0) absence of gradient (Ramphastos tucanus), (1) presence of gradient (Aechmophorus occidentalis), (2) binary gradient (Sarcoramphus papa), (3) inverse gradient (Fluvicola nengeta). (PDF) [file pone.0059451.s004.pdf]
